# Decreased Macular Retinal Thickness in Patients With Pterygium

**DOI:** 10.3389/fneur.2022.881190

**Published:** 2022-06-01

**Authors:** Feng Wang, Li Qi Liu, Rong Bin Liang, Li Juan Zhang, Hui Ye Shu, Xu Lin Liao, Yi Cong Pan, Jie Li Wu, Ting Su, Yi Shao

**Affiliations:** ^1^Department of Ophthalmology, Meizhou Pepole's Hospital, Meizhou, China; ^2^Department of Ophthalmology, First Affiliated Hospital of Nanchang University, Nanchang, China; ^3^Department of Ophthalmology and Visual Sciences, The Chinese University of Hong Kong, Shatin, Hong Kong SAR, China; ^4^Department of Ophthalmology, Xiang'an Hospital of Xiamen University, Xiamen, China; ^5^Department of Ophthalmology, Massachusetts Eye and Ear Infirmary, Harvard Medical School, Boston, MA, United States

**Keywords:** pterygium, optical coherence tomography angiography, macular, retinal thickness, examine

## Abstract

**Purpose:**

To explore alterations in macular retinal thickness (RT) and analyze correlation between macular RT and pterygium area, length in pterygium patients.

**Methods:**

Totally 13 patients with pterygium (left eye) and 13 healthy controls (left eye) were recruited. OCTA was applied to scan each eye to generate three-dimensional images. Based on the Early Treatment Diabetic Retinopathy Study (ETDRS) method, each image was divided into nine subregions for the ETDRS: central (C); inner superior (IS); outer superior (OS); inner nasal (IN); outer nasal (ON); inner inferior (II); outer inferior (OI); inner temporal (IT); and outer temporal (OT). The macular RT in each subregion was measured. Furthermore, the correlation between RT and the area, length of pterygium was analyzed.

**Results:**

The visual acuity of pterygium patient was different from that of the control (*P* < 0.05). Besides, decreased intraretinal thickness of the IN and ON, increased intraretinal thickness of OT, decreased extraretinal thickness of OT, IN, ON, OS, and decreased retinal full layer thickness of medial superior, OS, IN, ON, and II subregions in pterygium group were observed. There was a negative correlation between RT of the IN and ON subregions and the length of pterygium (r = −0.5803 and r = −0.6013, *P* = 0.0376 and *P* = 0.0297). The RT of IN subregion was negatively correlated with pterygium area (r = −0.5844, *P* = 0.0359). According to the receiver operating characteristic analysis, in the ON subregion, the areas under the curve of the inner retinal thickness, outer retinal thickness and the whole retinal thickness were 1.0 (95% CI: 1.0), 0.882 (95% CI: 0.715 and 0.963), and 1.0 (95% CI: 1.0). The smallest area under the curve of retinal thickness in OT subregion was 0.018 (95% CI: 0–0.059).

**Conclusion:**

RT of pterygium patients was significantly decreased, and the main alterations occurred in the temporal side suggesting there might exist retinal structural alterations in pterygium.

## Introduction

Pterygium is an ocular disorder occurring on the surface of the eye. The main characteristic is the pterygoid growth which could invade the limbus and conjunctiva on the adjacent cornea (1). The prevalence of pterygium ranges from 3 to 19.5% (2); although it typically occurs in the lateral nasal position, it occasionally develops in both directions (3). The pathogenesis of pterygium is currently unclear; however, research suggests that environmental factors, especially ultraviolet radiation (UV), play an important part (3, 4). Surgical resection is the only effective treatment for pterygium. However, depending on the technology used, the risk of recurrence can be as high as 89% (3). Therefore, the long-term efficacy of surgery is debatable. Corneal irregularity has an important influence on maintaining good eyesight after surgery. The improvement will not be evident in the early postoperative period. Hence, the evaluation of visual function has important clinical significance in determining whether surgery should be performed (5). Nevertheless, the human fundus macular area is responsible for almost the entire vision of humans and contains the highest distribution of visual (6) and retinal ganglion cells.

Recent studies have shown that changes in the anterior segment can also cause changes in macular structure. Zhang et al. (7) found that in the unilateral congenital cataract patients, the results of OCTA recorded after cataract surgery revealed increased vascular density and retinal thickness (RT), compared with that recorded before surgery. These findings suggested that the refractive axis became transparent after alleviating visual deprivation in the affected eyes would increase the light stimulation to fundus and change the structure. This increases the amount of light signal stimulation that enters the eye. Srujana et al. (8) used OCT to analyze 67 cases of keratoconus (129 eyes) and 87 cases of non-keratoconus (174 eyes). The average RT of the central fovea and macular area of keratoconus patients was lager than that of the control. They hypothesized that macular changes may be related to broader eye structural disorders of patients with keratoconus or optimization of the compensation mechanism of corneal irregular vision. Extension of pterygium beyond 2.2 mm results in obvious corneal astigmatism (9). Corneal astigmatism (mainly regular) or direct invasion of the visual axis will lead to visual problems (10). As a non-invasive *in vivo* imaging technique, OCTA can display morphological and quantitative information regarding fudus alterations in detail (11), including microvascular system of the retina and choroid (12), which is helpful to diagnose of myopia (13), microvascular changes in macular area after Phacoemulsification (14), glaucoma (15), thyroid-associated ophthalmology (TAO) (16) etc. Thus far, OCTA has not been used to study whether macular area changes is related to pterygium or not. In this study, we used OCTA to analyze the macular retina of all subjects and investigate changes of RT. The correlation between macular RT and pterygium length and area in patients with pterygium was also evaluated.

## Materials and Methods

### Research Subjects

This retrospective cross-sectional study was carried out at The First Affiliated Hospital of Nanchang University (Nanchang, China) in 2021. All subjects were recruited from the Outpatient Department and examined by the same retina specialist and divided into two groups. Patients diagnosed with pterygium belong to the experimental group and healthy subjects matched for sex and age with no ocular or systemic disease constitute the control.

### Recruitment Criteria

A total of 26 subjects, including 13 patients with primary pterygium in the left eye (pterygium group; aged 40–52 years; mean age: 46 ± 3 years) and 13 healthy individuals (control group; 13 left eyes; mean age: 45 ± 4 years) were recruited. The clinical condition score was evaluated using the NOSPECS classification, and inflammation score would be determined through the 7-point Clinical Activity Score. The control group included healthy subjects without ocular abnormalities according to clinical examination at the medical center and the OCTA imaging examination did not display any disorder.

Inclusion criteria (1) Patient who was diagnosed with pterygium in left eye for the first time (2) The primary pterygiod growth occurred in the nasal side; (3) A medical history of 7 months to 20 years, with an average of 8.77 ± 5.94 years; (4) pterygium invasion toward the corneal limbus is almost 2.0–7.0 mm; (5) binocular visual acuity was in the normal range (without anisometropia >2 D); (6) the intraocular pressure ranged from 10 to 21 mmHg; (7) Fundus examination did not display obvious abnormality.

Exclusion condition (1) the medical history of ocular trauma and surgery; (2) drug treatment or radiotherapy within the recent 14 days; (3) anterior abnormality, such as corneal diseases (including patients who wear corneal contact lens, pseudopterygium and fibromyalgia), glaucoma, cataract; (4) retinal abnormality, such as highly myopic, maculopathy and retinal detachment (5) systemic diseases which can affect ocular vasculature, such as diabetes, hypertension, nephropathy; (6) patients with amblyopia; (7) patients with systemic diseases such as autoimmune diseases and nervous diseases; (8) pregnancy and lactating women; (9) Pupil dilation and patients who are sensitive to mydriatic. The general condition of the patients and condition of pterygium are shown in Table 1.

**Table 1:** Demographic characteristics and clinical findings of patients with Pterygium and HCs.

**Condition**	**Pterygium**	**HC**	**t**	***p*** -Value^*^
Age(years)	46 ± 3	45 ± 4	0.40	0.69
Sex(male/female)	2/11	2/11	N/A	>0.99
uncorrected visual acuity	0.6 ± 0.1	0.9 ± 0.1	0.39	0.04*
Best-corrected visual acuity	0.7 ± 0.1	1 ± 1	0.35	0.04*
SE-L(diopter)	1.15 ± 1.00	1.25 ± 0.75	0.19	0.83
Astigmatism -L(diopter) AL(mm)	2.75 ± 0.75 23.53	1.25 ± 0.50 23.72	4.17 0.12	0.03* 0.89
SSI	9 ± 1	9 ± 1	N/A	>0.99
The course of pterygium(years)	8.77 ± 5.94	/	/	/
The Length of pterygium(mm)	5.24 ± 0.89	/	/	/
The height of pterygium(mm)	5.84 ± 1.20	/	/	/
The area of pterygium(mm)	16.62 ± 5.72	/	/	/
Heart rate(beats/min)	70 ± 6	69 ± 7	−0.13	0.98
Systolic blood pressure(mm Hg)	124 ± 6	123 ± 10	−0.25	0.89
Diastolic blood pressure(mmHg)	82 ± 7	83 ± 6	−0.12	0.73
Mean intra-ocular pressure(mmHg)	15 ± 2	14 ± 3	−0.25	0.81

* *P < 0.05 Independent t-tests comparing two groups*.

*HC, healthy control; N/A, not applicable; L, left; SE, Spherical equivalent; AL, axial length; SSI, Signal strength Index*.

### Ethical Considerations

The study was carried out in accordance with the principles of the Helsinki Declaration seriously and was approved by the Medical Ethics Committee of Nanchang University. Before the consent forms were signed, all subjects had comprehended the objectives and methods.

### Clinical Examinations

Volunteers completed the following testings.

**Figure 1:**
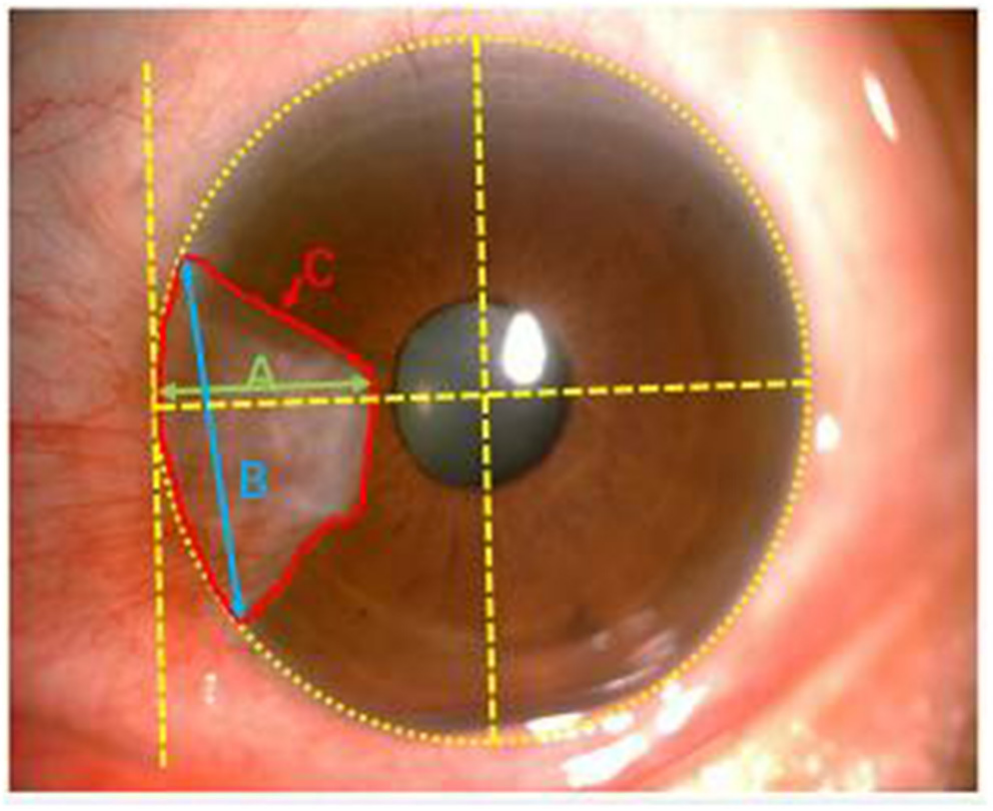
Note clinical photographic imaging using Zeiss slit lamp mounted camera and nihimage J software to measure the size of pterygium. Green, blue and red lines depict length (A), height (B) and area (C), respectively.

(1) Slit lamp examination. The anterior segment of the eye was examined by Slit lamp microscopy including ocular surface inflammation, cataract with severe intraocular astigmatism, the pterygium size;

(2) Optometry. Obtain corrected binocular visual acuity of all participants by standard logarithmic visual acuity chart;

(3) Intraocular pressure (IOP). Measure intraocular pressure by transpalpebral diatom tonometer (TDT, BiCOM, Long Beach, NY, USA) for 3 times and calculatethe average values. IOP recorded of both eyes are in the normal range. The D-value between the different measurements of the same one was ≤ 3 mmHg.

(4) Calculate the size of pterygium by ImageJ software (WayneRasband,NIH,USA). The horizontal distance between corneal limbus and pterygium head is length. The distance between upper and lower point where pterygium grows into the limbus is the height, and coverage of pterygium to cornea is the area (Figure 1) (17). Measure the length (A), height (B) and area (C) of each meat for 5 times, and calculate the average.

### OCTA Imaging

**Figure 2:**
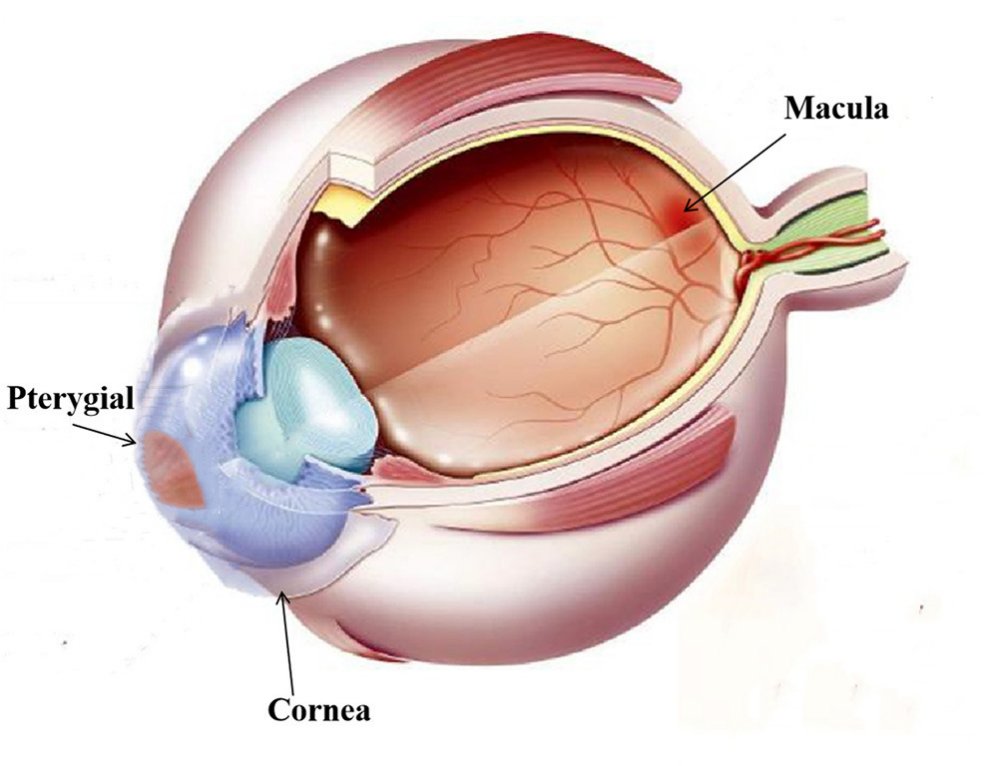
Illustration of the pterygium and macular retinal thickness area: As shown in the figure, see the pterygium of the nasal limbus of the left eye and the macula of the fundus.

**Figure 3:**
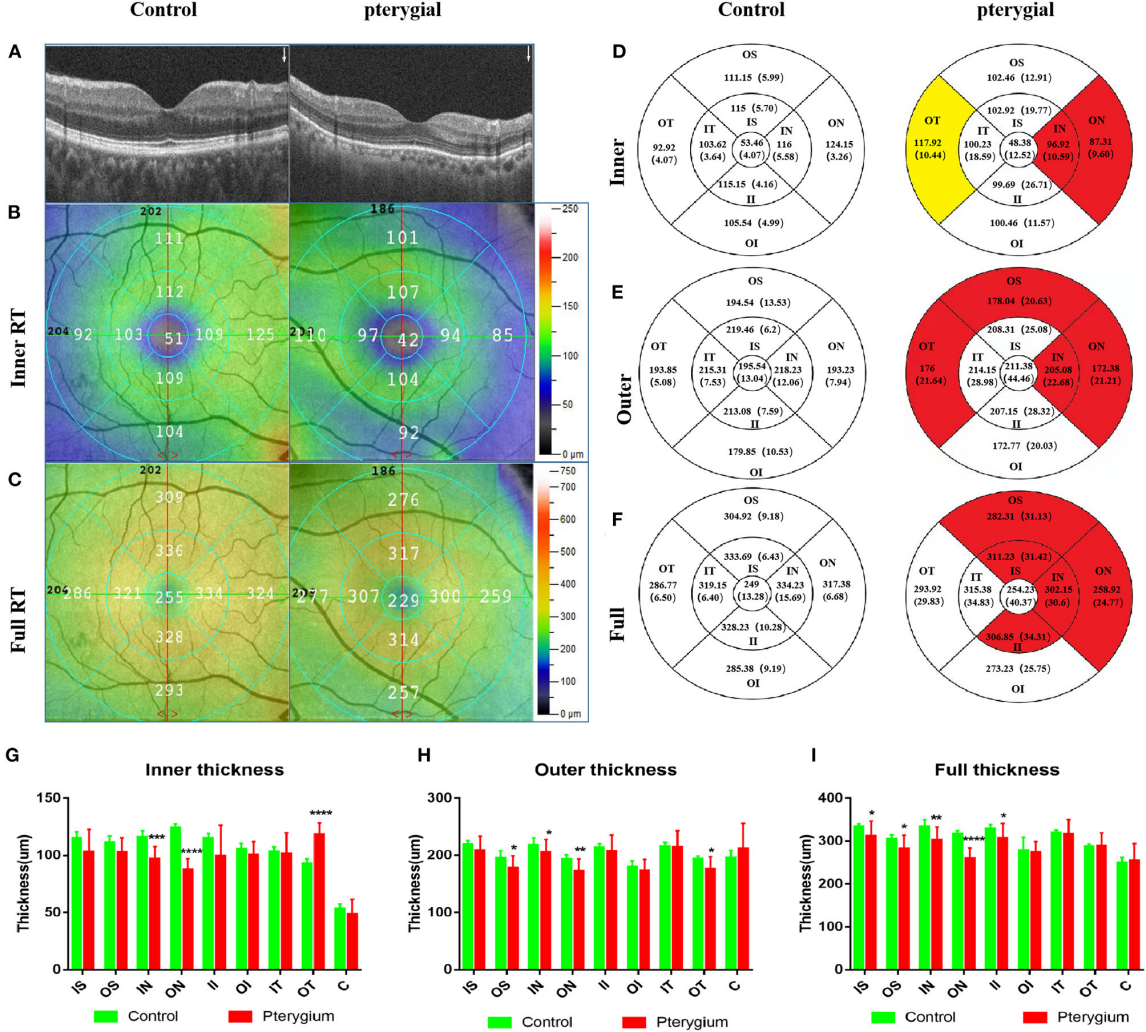
OCTA images and RT analysis of the control group and pterygium group. **(A)** RT cross sections of the control group and pterygium group measured by OCTA. **(B)** Internal RT was measured with ETDRS. **(C)** Complete RT measurement diagram. **(D)** Internal RT measurement results of the control group and pterygium group (red area indicates the area with significant decrease in retinal thickness, yellow area indicates the area with significant increase in retinal thickness, *P* < 0.05). **(E)** External RT measurements in the control group and pterygium group **(F)** complete RT measurements. **(G–I)** Analysis of retinal thickness results in the control group and pterygium group. OCTA, optical coherence tomography angiography; RT, retinal thickness; ETDRS, early treatment of diabetic retinopathy study; IS, inner superior; OS, outer superior; IN, inner nasal; ON, outer nasal; II, inner inferior; OI, outer inferior; IT, inner temporal; OT, outer temporal; C, center. *P < 0.05; **P < 0.01; ***P < 0.001; ****P < 0.0001.

We used RTVue Avanti XR system to performed OCTA imaging and display both the cross-section of the retina and microvasculature. The scanning speed was set at 70,000 A-scans/s; the axial resolution was 5 mm; the horizontal resolution was 22 μm; the central wavelength was 840 nm; and the bandwidth was 45 nm. The imaging time was 3.9 s. Five angiograms were performed in the 3 × 3 mm scan mode, focusing on the fovea along the x axis (total: 216 A-scans) and along the y axis (216 raster positions per scan). At 216 y positions × 5 positions, we captured 1,080 B-scans at a speed of 270 frames (33 frames) pers. Following four volume scans, 6 × 6 mm OCTA images were obtained. Also, the 3 × 3 mm macular retinal image of each eye was obtained. According to the ETDRS, retina of each eye was divided into 9 subregions. Three cercles concentriques composed them. The radii were set at 0.5, 1.5 and 3 mm, respectively. These subregions whose thickness was analyzed were as follows: outer superior (OS); outer temporal (OT); outer nasal (ON); outer inferior (OI); inner superior (IS); inner temporal (IT); inner nasal (IN); inner inferior (II) and central (C); Each retinal layer includes the inner retina and the full-year retina. The relationship between pterygium and the retinal fovea is illustrated in Figure 2. The partition of macular retinal area is shown in Figures 3A–C.

### Statistical Analysis

All data were analyzed by statistical software package (statistica, v7.1; StatSoft, Inc, Tulsa, OK, USA) and medcalc software (V10; medcalc software, mariakerke, Belgium). Continuous variables are proposed to be expressed as mean ± standard deviation (SD). RT of each subregion among each group was analyzed by One Way ANOVA. The difference between two group was access from minimum significant difference by a specific test. *P* < 0.05 was statistically significant. Graphpad prism 7.0 was used to analyzed the correlation between retinal thickness and the length, width and area of pterygium. Use Graphpad prism 7.0 to analyze the relation between RT and pterygium size, and the Receiver Operator Characteristic (ROC) curves of retinal thickness in the retinal epithelium was plotted by SPSS 23.0 (IBM Corp, Armonk, NY, USA) to differentiate between patients with pterygium and healthy subjects.

## Results

### Macular Retinal Thickness

Regional retinal thicknesses of the experimental and the control is shown in Table 2. The internal RT in the IN and ON subregions of patient were lower than that recorded in the control (*P* = 0.0001, *P* < 0.0001, respectively). The internal RT of the OT subregion in the patient with pterygium was higher than that recorded in the healthy (*P* < 0.0001) (Figures 3D,G). Three other domains of the inner loop (superior part: *P* = 0.0703; lower part: *P* = 0.0673; temporal part: *P* = 0.6463, respectively), two subregions of the outer loop (superior part: *P* = 0.0547; lower part: *P* = 0.1543, respectively), C subregions (*P* = 0.1921) were not distinctly different when comparing those two groups.

**Table 2:** Comparison of macular retinal thickness at different locations between patients with Pterygium and health controls.

**Location**	**Pterygium (*n* = 13,13 eyes)**	**HC (*n* = 13,13 eyes)**	***p*** **-Value^**a**^**
Macular inner retinal thickness (μm, mean ± SD)				
IS	115.00 ± 5.70	102.92 ± 19.77	0.0703
OS	111.15 ± 5.99	102.92 ± 19.77	0.0547
IN	116.00 ± 5.58	96.92 ± 10.59	**0.0001**
ON	124.15 ± 3.26	87.31 ± 9.60	**<0.0001**
II	115.15 ± 4.16	99.69 ± 26.71	0.0673
OI	105.54 ± 4.99	100.46 ± 11.57	0.1543
IT	103.62 ± 3.64	100.23 ± 18.59	0.6463
OT	92.92 ± 4.07	117.92 ± 10.44	**<0.0001**
C	53.46 ± 4.07	48.38 ± 12.52	0.1921
Macular outer retinal thickness (μm, mean ± SD)				
IS	219.46 ± 6.20	208.31 ± 25.08	0.1366
OS	194.54 ± 13.53	178.04 ± 20.08	**0.0454**
IN	218.23 ± 12.06	205.08 ± 22.68	**0.0268**
ON	193.23 ± 7.94	172.38 ± 21.21	**0.0062**
II	213.08 ± 7.59	207.15 ± 28.32	0.4172
OI	179.85 ± 10.53	172.77 ± 20.03	0.2649
IT	215.31 ± 7.53	214.15 ± 28.98	0.8775
OT	193.85 ± 5.08	176.00 ± 21.64	**0.0180**
C	195.54 ± 13.04	211.38 ± 44.46	0.2837
Macular full retinal thickness (μm, mean ± SD)				
IS	333.69 ± 6.43	311.23 ± 31.42	**0.0398**
OS	304.92 ± 9.18	311.23 ± 31.42	**0.0283**
IN	334.23 ± 15.69	302.15 ± 30.6	**0.0014**
ON	317.38 ± 6.68	258.92 ± 24.77	**<0.0001**
II	328.23 ± 10.28	306.85 ± 34.31	**0.0363**
OI	285.38 ± 9.19	273.23 ± 25.75	0.1476
IT	319.15 ± 6.40	315.38 ± 314.83	0.6908
OT	286.77 ± 6.50	293.92 ± 29.83	0.4465
C	249.00 ± 13.28	254.23 ± 34.31	0.6825

*Bold values indicates P < 0.05*.

a *Generalized estimating equation models were used to obtain P-values comparing mean inner, outer and full macular retinal thickness between Pterygium patients and healthy subjects. Models were adjusted for age, intraocular pressure, acuity, blood pressure*.

*HC, healthy control; IS, inner superior; OS, outer superior; IN, inner nasal; ON, outer nasal;II, inner inferior; OI, outer inferior; IT, inner temporal; OT, outer temporal; C, center*.

The external retinal thicknesses in the IN, ON, OT, and OS subregions of pterygium were lower than that measured in the healthy (*P* = 0.0268, *P* = 0.0062, *P* = 0.0180, *P* 0.0454) (Figures 3E,H). The other three subregions of the inner loop (temporal part: *P* = 0.8775; lower part: *P* = 0.4172; superior part: *P* = 0.1366), one subregion of the outer ring (lower part: *P* = 0.2649), C subregion (*P* = 0.2837) were not distinctly different when comparing those two groups.

The full-layer retinal thickness of the IN, ON, IS, II, and OS subregions in the pterygium group was lower than that noted in the control (*P* = 0.0014, *P* < 0.0001, *P* = 0.0398, *P* = 0.0363, *P* = 0.0283, respectively) (Figures 3F,I). One other subregion of the inner ring (nose: *P* = 0.0283), two subregions of the outer ring (lower part: *P* = 0.1476; temporal: *P* = 0.4465), and the foveal center (*P* = 0.6825) were not distinctly different between when comparing those two groups.

### ROC Curve Analysis of RT

**Figure 4:**
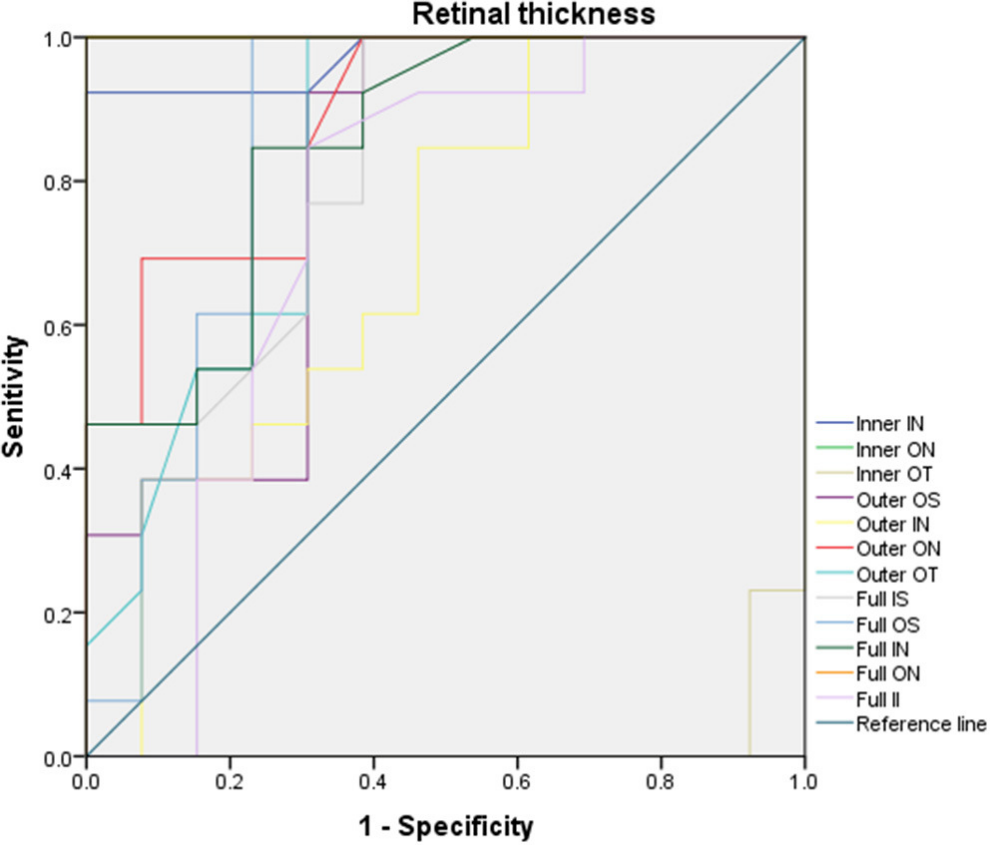
ROC curve analysis of RT. In ON subregion, the areas under the curve of the inner retinal thickness, outer retinal thickness and the whole retinal thickness were 1.0 (95% CI: 1.0), 0.882 (95% CI: 0.715–0.963), and 1.0 (95% CI: 1.0). In the IN area, the areas under the curve of external retinal thickness and full retinal thickness were 0.973 (95% CI: 0.916–1.0), 0.698 (95% CI: 0.491–0.905), and 0.852 (95% CI: 1.0), respectively, the minimum area under ROC curve of RT in OT area is 0.018 [95% confidence interval (CI): 0–0.059]. ROC, receiver operating characteristic; CI, confidence interval; RT, retinal thickness; IS, inner superior; OS, outer superior;IN, inner nasal; ON, outer nasal; II, inner inferior; OI, outer inferior; IT, inner temporal; OT, outer temporal; C, center.

Use OCTA results to evaluate the sensitivity and specificity of RT to changed anterior segments of pterygium patients (Figure 4). In ON subregion, the areas under the curve of the inner retinal thickness, outer retinal thickness and the whole retinal thickness were 1.0 (95% CI: 1.0), 0.882 (95% CI: 0.715–0.963), and 1.0 (95% CI: 1.0). It suggested medium to high specificity and sensitivity in pterygium diagnosis (Figure 4). In the IN area, the areas under the curve of external retinal thickness and full retinal thickness were 0.973 (95% CI: 0.916–1.0), 0.698 (95% CI: 0.491–0.905), and 0.852 (95% CI: 1.0), respectively. That indicated medium to high specificity and sensitivity in pterygium diagnosis (Figure 4). The minimum area under the ROC curve of retinal thickness in OT area was 0.018 (95% CI: 0–0.059), indicating low diagnostic specificity and sensitivity for pterygium, with high possibility for false positive results (Figure 4).

### Correlation Between Retinal Thickness and Length and Area of Pterygium

**Figure 5:**
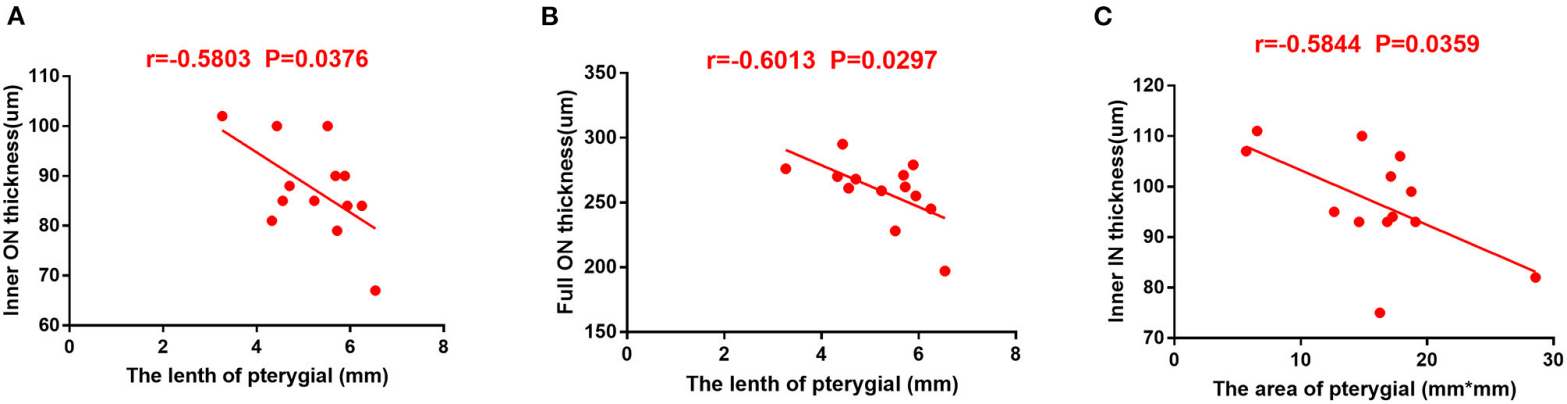
The correlation between RT and pterygium length and area in patients with pterygium. **(A)** RT in ON area of internal retina was in a negative correlation with the length of pterygium (r = −0.5803, *P* = 0.0376). **(B)** RT in ON region of the complete retina was in a negative correlation with area of pterygium (r = −0.6013, *P* = 0.0297). **(C)** Total retinal RT is negatively correlated with pterygium area (r = −0.5844, *P* = 0.0359). RT, retinal thickness; IN, inner nasal; ON, outer nasal.

There was a negative correlation between RT of the IN and ON subregions and the length of pterygium (r = −0.5803 and r = −0.6013, *P* = 0.0376 and *P* = 0.0297), indicating that a lower RT was associated with longer pterygium (Figures 5A,B). In the pterygium group, RT in the IN region was in a negative correlation with pterygium area (r = 0.5844, *P* = 0.0359). This revealed that lower RT is related to larger pterygium (Figure 5C).

## Discussion

In this study by using the OCTA approach, our data revealed significant alterations in RT of patients with pterygium. According to ROC curve, the inner, the outer, and full-layer retinal thickness of the IN and ON regions of patients with pterygium exhibited high sensitivity and specificity, whereas the ON region showed low sensitivity and specificity of the approach. These findings indicated that the RT of individuals with pterygium was distinctly decreased, and alterations mainly occurred on the temporal side. At the same time the area and length of pterygium were in a negative correlation with retinal thickness. This is the first time to investigate retinal thickness alterations in pterygium patients. The research provides a potential way to assess the progression of pterygium according to the changed retinal thickness. Also, it may indicate the natural etiology of pterygium at the retinal level.

However, it is unclear whether the alterations in RT occurs due to alterations in the anterior segment, which occures prior to these alterations or accompanied by alterations in the anterior segment. Nevertheless, we hypothesized several possible mechanisms underlying the alterations. This alteration in RT may be related to the retinal movement of photoreceptors, similar to those observed in animal models of visual deprivation. Liang et al. (18) used opaque occluders to cover one eye of hatchling chicks reared for 1, 2 or 4 weeks to expound the pathogenesis of deprivation myopia. Thinner retina and choroid existed in all deprived eyes. Thicker cone inner segments, rod outer segments, damaged cone outer segment made their distal tips can attach to the RPE basement membrane or depress the nucleus. In this study, the visual acuity of subjects with pterygium was decreased (Table 1). We hypothesized the “rod-push” mechanism induces thinner retina with poor vision. Pterygium may cover the cornea on the nasal side, resulting in thickening of the corneal tissue. The decrease in the thickness of the temporal retina may occur as a compensatory response to corneal alterations and prevent the overall structural disorder of the eye. Feng et al. (19) indicated total macular volume could increase after corneal refractive surgery while Lasik should not influence fundus. Therefore, according to OCTA scans, alterations in the optical surface of the eye may lead to retina changes. Longer length and larger area of pterygium will result in more compensation for the apparent volume of the retina and lower thickness of the temporal retina. This is consistent with our study results, implying a compensation mechanism in the eye, through which alterations in one component might impact other components.

Another possible reason for alterations in RT is a decrease in the amount of light. It is reported that cataract could block 18–40% of different wavelengths of light (20). Zhou et al. (21) found that macular vascular density increased after cataract surgery. Although some studies have shown that inflammation after cataract surgery is a potential pathogenic factor for fundus alterations after cataract surgery (22), inflammation itself cannot last for prolonged periods of time. The investigators suggested that higher light levels could induce retinal metabolism and angiogenesis; this process can be taken as postoperative phototoxicity and explain why age-related macular degeneration is more common in intraocular lens eye (23). Similarly, pterygium on the side of the nose will also block light. Nevertheless, whether the decrease in light will also lead to a decrease in the activity and metabolic needs of photoreceptor cells in the retina, thereby resulting in a decrease in blood flow (24), will still need further investigations.

**Figure 6:**
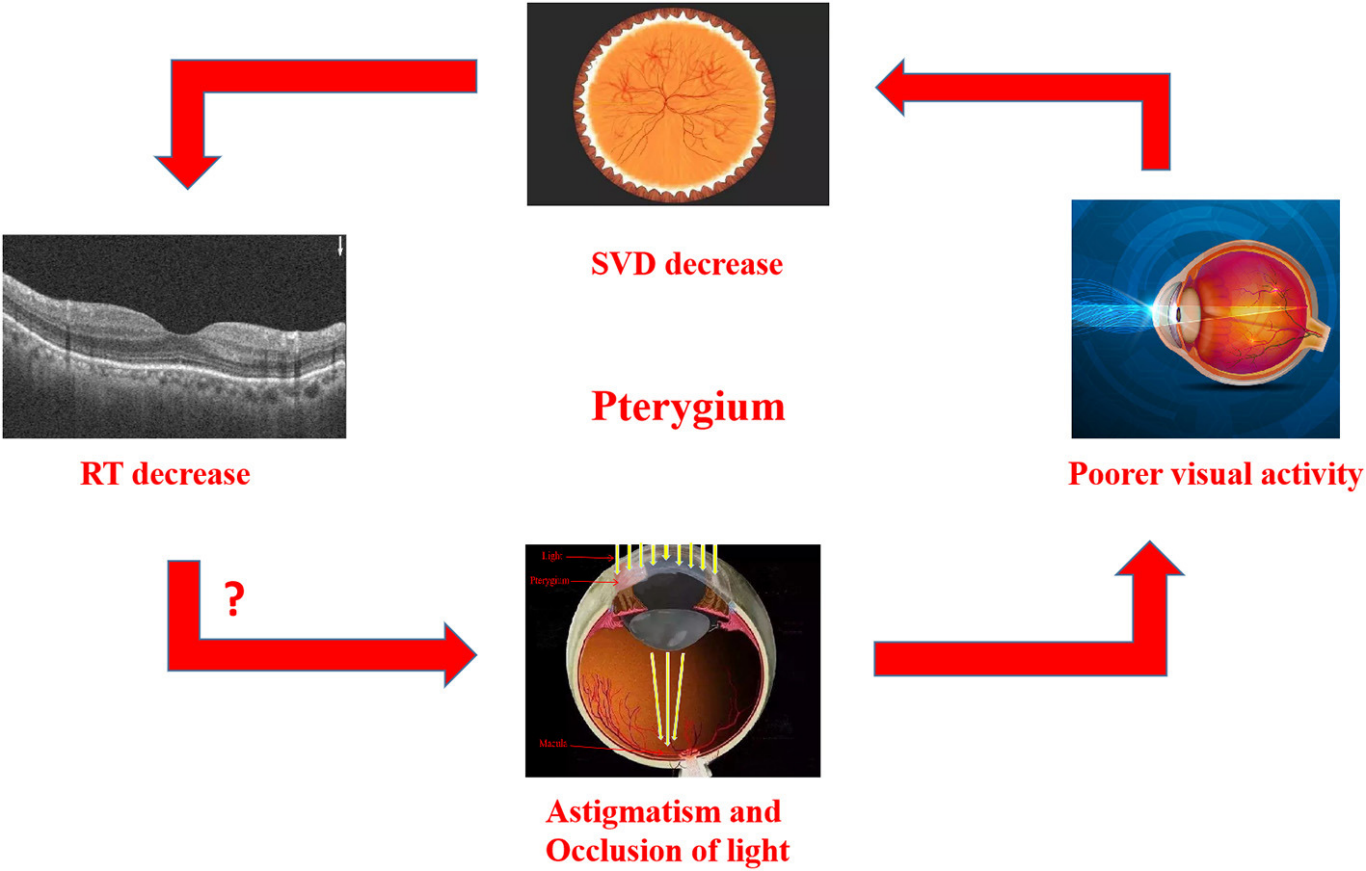
Relationship between RT thinning in patients with Pterygium, and there is also a correlation between RT thinning and visual impairment. In Pterygium group, astigmatism and occlusion of light may lead to the decrease of vision., SVD in macular and RT in related areas. The macular area is marked on the image of eyeball, which reveals the decrease of SVD and RT in this area. RT, retinal thickness; SVD, superficial vessel density.

In our previous study, there was also a decrease in vascular density (VD) in temporal subregion of pterygium (25). Retina and brain exhibit similar metabolic activities because they derive from the same embryonic tissue. The lack of blood supply could induce retinal cell apoptosis (26). A reduction in the blood supply to the temporal side of the retina may lead to not only chronic ischemia, but also the death of rods and cones due to reduced energy supply and loss of vision (26). Thus, all these alterations lead to a decrease in RT. which is supported by our findings. Our results also demonstrated that alterations in the internal and panretinal thickness of the ON subregion were in a distinctly negative correlation with length of pterygiod growth while the intraretinal thickness of the IN subregion was in a distinctly negative correlation with the area. Han et al. (27) reported that the corneal astigmatism caused by pterygium was more correlated with the length than with width and area. Mohammad-Salih and Sharif (9) reported the corneal astigmatism was most correlated with the dilatation of pterygium, then the whole area This is consistent with our results showing the length and area of pterygium have the greatest impact on corneal astigmatism, which thus affects vision and further leads to alterations in fundus RT. On the other hand, as light vision is done by cone cells, reducing light leads to a decrease of cellular activity. While the location of the ON subregion is shown to be closest to the central fovea, the consistency in location supports that the decrease in light may reduce macular blood vessel density. Accordingly, a larger size or longer length of pterygium, will result in more light block, thus lead to more significant alterations, which further confirms our results (Figure 6).

Certain shortcoming existed in this research. First of all, the sample size was not large. And then, the optometry of pterygium patients was lower than that of healthy individuals (Table 1). This might be explained that pterygium could cause corneal alterations (28), thus leading to poor visual acuity. Besides, the large pterygium will invades the pupil area; thus result in a decrease in visual acuity. Therefore, the effect of alteration in RT on visual acuity warrants further investigation. In addition, in this study, we obtained false positive results of increased thickness of the retina in the nasal OT subregion of patients with pterygium. As a non-invasive technique, OCT could use light to scan retina or anterior segment and obtain image according to the interference with backscatter or reflectance (29). While, depend on the enface and depth-encoded slabs, OCTA technology can display flowing image of retinal and choroidal vascular plexus, which would be indistinct with previous imaging mode (30). However, OCTA also has its own shortcomings, e.g., Opaque medium limits OCTA examination and lead to signal abnormality because light source is the foundation of the technology. Significant astigmatism also affects the assessment of RT and results in signal attenuation and shadow artifacts (12). Hence, we speculate this may be related to pterygium, which blocks the light on the side of the nose. Consequently, motion artifacts or measurement errors occur, resulting in an increase in the thickness of the retina in the measured OT area.

## Conclusion

In summary, the RT of patients with pterygium is decreased than that of healthy individuals, and the main alteration is observed in the temporal side. The results show that, there are important differences in the posterior segment of pterygium as well as the anterior segment. Therefore, macular alterations may be related to broader eye structural disorders in patients with pterygium or the optimization of the compensation mechanism of irregular corneal vision and light occlusion in patients with pterygium. However, it is unclear whether these alterations occur before or as a result of the development of pterygium. Further investigation is warranted to repeat the present findings and determine their clinical importance.

## Data Availability Statement

The original contributions presented in the study are included in the article/supplementary material, further inquiries can be directed to the corresponding author.

## Ethics Statement

This study confirmed to the Declaration of Helsinki and had formal approval from the Medical Ethics Committee of the First Affiliated Hospital of Nanchang University. The patients/participants provided their written informed consent to participate in this study. Written informed consent was obtained from the individual(s) for the publication of any potentially identifiable images or data included in this article.

## Author Contributions

FW: acquisition, analysis, interpretation of data, and drafting the article. LL: acquisition and interpretation of data. RL: acquisition and analysis of data. LZ, HS, and XL: literature revision and data discussion. YP: acquisition of data. JW: literature revision. TS: data discussion. YS: conception, design of the study, and supervised the work. All authors critically revised the manuscript for important intellectual property and approved the final version to be submitted.

## Funding

This work was supported by National Natural Science Foundation (No: 82160195); Central Government Guides Local Science and Technology Development Foundation (No: 20211ZDG02003); Key Research Foundation of Jiangxi Province (Nos: 20181BBG70004 and 20203BBG73059); Excellent Talents Development Project of Jiangxi Province (No: 20192BCBL23020); Natural Science Foundation of Jiangxi Province (No: 20181BAB205034); Grassroots Health Appropriate Technology “Spark Promotion Plan” Project of Jiangxi Province (No: 20188003); Health Development Planning Commission Science Foundation of Jiangxi Province (Nos: 20201032 and 202130210); Health Development Planning Commission Science TCM Foundation of Jiangxi Province (Nos: 2018A060 and 2020A0087). Research and Cultivation Project of Guang-dong Meizhou people's Hospital (PY-2021060).

## Conflict of Interest

The authors declare that the research was conducted in the absence of any commercial or financial relationships that could be construed as a potential conflict of interest.

## Publisher's Note

All claims expressed in this article are solely those of the authors and do not necessarily represent those of their affiliated organizations, or those of the publisher, the editors and the reviewers. Any product that may be evaluated in this article, or claim that may be made by its manufacturer, is not guaranteed or endorsed by the publisher.
